# Sequential Cross-Species Chromosome Painting among River Buffalo, Cattle, Sheep and Goat: A Useful Tool for Chromosome Abnormalities Diagnosis within the Family Bovidae

**DOI:** 10.1371/journal.pone.0110297

**Published:** 2014-10-17

**Authors:** Alfredo Pauciullo, Angela Perucatti, Gianfranco Cosenza, Alessandra Iannuzzi, Domenico Incarnato, Viviana Genualdo, Dino Di Berardino, Leopoldo Iannuzzi

**Affiliations:** 1 Institute for Animal Production System in Mediterranean Environment, National Research Council, Naples, Italy; 2 Department of Agriculture, University of Naples Federico II, Portici, Italy; University of Science and Technology of China, China

## Abstract

The main goal of this study was to develop a comparative multi-colour Zoo-FISH on domestic ruminants metaphases using a combination of whole chromosome and sub-chromosomal painting probes obtained from the river buffalo species (*Bubalus bubalis*, 2n = 50,XY). A total of 13 DNA probes were obtained through chromosome microdissection and DOP-PCR amplification, labelled with two fluorochromes and sequentially hybridized on river buffalo, cattle (*Bos taurus*, 2n = 60,XY), sheep (*Ovis aries*, 2n = 54,XY) and goat (*Capra hircus*, 2n = 60,XY) metaphases. The same set of paintings were then hybridized on bovine secondary oocytes to test their potential use for aneuploidy detection during *in* *vitro* maturation. FISH showed excellent specificity on metaphases and interphase nuclei of all the investigated species. Eight pairs of chromosomes were simultaneously identified in buffalo, whereas the same set of probes covered 13 out 30 chromosome pairs in the bovine and goat karyotypes and 40% of the sheep karyotype (11 out of 27 chromosome pairs). This result allowed development of the first comparative M-FISH karyotype within the domestic ruminants. The molecular resolution of complex karyotypes by FISH is particularly useful for the small chromosomes, whose similarity in the banding patterns makes their identification very difficult. The M-FISH karyotype also represents a practical tool for structural and numerical chromosome abnormalities diagnosis. In this regard, the successful hybridization on bovine secondary oocytes confirmed the potential use of this set of probes for the simultaneous identification on the same germ cell of 12 chromosome aneuploidies. This is a fundamental result for monitoring the reproductive health of the domestic animals in relation to management errors and/or environmental hazards.

## Introduction

One of the main goals of cytogeneticists is the characterization of chromosomes by simple, rapid and reliable approaches. In this regard, the classical banding techniques are still the most used procedures since they represent standard and well established karyotyping methods. This is particularly true for the farm animal populations, whose routine cytogenetic analysis has been performed mainly by the application of classical methods [1].

Despite their wide application, several technical restrictions characterize the classical banding techniques [2] among which the size variations in a chromosomal band or the chromosome itself require a deep knowledge of the banding pattern to resolve complex karyotypes. However, in the last decade, the development of molecular cytogenetic techniques based on fluorescence *in* *situ* hybridization (FISH) led to a significant improvement in the accuracy of cytogenetic investigation, representing a valid alternative to the standard methods. In humans, the achievement of 24 colour FISH-based karyotyping (M-FISH, SKY, COBRA) [3], [4], [5] was the culmination of this technological progress.

Further advancements were reached in humans with chromosome arm-specific [4], [6], region-specific [7], [8], centromeric [9] and sub-telomeric probe sets [10], [11], until arriving to the recent karyo-mapping [12] which offers a very fine clinical investigation for chromosome imbalances and miscarriage detections.

The applications of FISH techniques in farm animals and humans are very similar and approximately the same level of advancement was reached from animal cytogeneticists in the last two decades. The use of chromosome paintings and DNA probes in domestic animals allowed several important questions to be resolved, including a) detection of chromosome aberrations and complex karyotypes; b) gene mapping and comparative mapping; c) identification of conserved syntenic blocks between species and d) description of chromosome evolution [13], [14].

Contrary to humans, the use of multi-colour FISH is still very limited in animal cytogenetics. This is mainly due to the lack of existing commercial probes which are essentially limited to sex chromosomes for most of the domestic species and to two autosomes in cattle for the detection of rob (1; 29) translocation. In addition, only some laboratories of excellence in the world have probes availability [14], thus limiting the application of the method to few research groups. For instance, Kubickova et al. [15] proposed a tri-colour FISH to resolve rob (14; 20) and rob (16; 20) translocations in cattle, later seven-colour FISH using a specific paint pool was used in camels to identify smaller chromosomes [16], whereas recently a pool of 13 chromosome-specific painting probes were used to develop a sequential multi-colour FISH in river buffalo to quickly identify submetacentric chromosomes and gonosomes [17].

The aim of this investigation was to use a combination of whole chromosome and sub-chromosomal painting probes derived from river buffalo (*Bubalus bubalis*, river type, 2n = 50,XY) in a comparative multi-colour Zoo-FISH study on domestic ruminants such as cattle (*Bos taurus* L., 2n = 60,XY), sheep (*Ovis aries* L., 2n = 54,XY) and goats (*Capra hircus* L., 2n = 60,XY) to develop the first comparative M-FISH karyotype. In addition, we report on the application of this pool of probes on bovine secondary oocytes as potential tool for aneuploidy detection during *in* *vitro* maturation.

## Materials and Methods

### Ethics statements

Procedures were in accordance with the ethical standards of the national ethics committee on research on animal science of 7th June 2011. All institutional and national guidelines for the care and use of laboratory animals were followed. The protocol was approved by the Committee on the Ethics of Animal experiments of the CNR-ISPAAM (Permit Number: 0000391-18/03/2014).

### Cell cultures

Peripheral blood cultures from eight clinically healthy adult males (two river buffalo bulls, two cattle bulls, two goat rams and two sheep rams) reared in southern Italy were performed following the method described by Iannuzzi and Di Berardino [1]. Four replicates for each sample were prepared according to the conventional cultures protocol and subjected to 20 min of colcemid (0.05 µg/ml) treatment, followed by centrifugation steps, hypotonic (KCl 75 mM) and fixative methanol/glacial acetic acid (3∶1) treatments.

### In vitro maturation of COCs and oocyte fixation

Ovaries were collected from two slaughtered bovine cows and transported to the laboratory within two hours. Cumulus-oocyte complexes (COCs) were collected through aspiration with 21-gauge needles, washed in TCM-199 (Sigma, USA), and examined on Petri dishes under a stereomicroscope. Only oocytes with several compact cumulus cell layers and good morphology were selected for the maturation procedure. Groups of oocytes selected from each donor were transferred into 50-mL droplets of maturation medium consisting of TCM-199+10% foetal bovine serum (Gibco), supplemented with 0.5 mg/mL follicle-stimulating hormone (FSH) (Sigma), 5 mg/mL luteinizing hormone (LH) (Sigma), covered with sterile mineral oil (Sigma) and allocated in a humidified atmosphere containing 5% CO_2_ in air at 39°C for 24 h.

After 24 h maturation, the COCs were incubated for a few minutes in a 1 mg/mL hyaluronidase solution (Sigma) to remove the cumulus cells, washed in Phosphate Buffered Saline (PBS), and exposed to a hypotonic sodium citrate solution (0.8% w/v) for 3 min, followed by KCl (75 mM) treatment for 3 min. The fixation was carried out using cold methanol/glacial acetic acid (1∶1) solution. Oocytes were individually fixed at the center of a pre-cleaned slide, air-dried, and kept at −20°C until analysis.

### Chromosome microdissection and painting probes preparations

For the production of probes via chromosome microdissection, the river buffalo fixed lymphocyte suspension was spread onto a pre cleaned 24×60 mm coverslip, air dried and then treated for GTG-banding. According to Pauciullo et al. [17], the probes corresponding to the biarmed pairs (from 1 to 5) were produced by dissecting out the centromeric area, to avoid unspecific repetitive amplification of the centromeric regions. The probe corresponding to the X chromosome was produced by dissecting the region Xq21–25, analogous to the Xcen region of the bovine chromosome [18]. The probes corresponding to chromosomes 18 and Y were produced by scraping the entire chromosomes.

Briefly, each micro-needle used for microdissection was broken in a 0.2 ml tube containing a collection buffer made of 5X Sequenase reaction buffer (Affimetrix, USA) and water in a final volume of 3.4 µl. On average 15 copies of the same chromosome were collected in the each tube. All tubes underwent to topoisomerase I (10 U/µl) treatment at 37°C for 30 min followed by enzyme inactivation at 95°C per 10 min. Highly processive chromosomal amplification was accomplished by degenerate oligonucleotide primer and sequenase ver. 2.0 DNA polymerase (Affimetrix) through a primary DOP-PCR reaction carried out at 94°C for 1 min, 30°C for 1 min and 37°C for 2 min. The enzyme was diluted according to the manufacture’s guidelines and added during the annealing step at every cycle of the reaction for the first 8 cycles. Further 40 cycles of PCR amplification were performed at 94°C for 1 min, 56°C for 1 min and 72°C for 2 min in a reaction volume of 50 µl made of 1X AmpliTaq buffer, 3.5 mM of MgCl_2_, 1 pmol of primer, dNTPs each at 200 µM, 2.5 U of Ampli*Taq* DNA Polymerase (Applied Biosystem).

Each probe was labelled separately by using a secondary DOP-PCR using 2 µL of products from the first reaction as template. Labelling scheme was performed according to Pauciullo et al. [17], with Spectrum Orange-dUTP and Spectrum Green-dUTP (Abbott, USA).

### Fluorescent in situ hybridization (FISH)

Six sequential rounds of FISH were performed on the same slide. Each round was realized by using two probes simultaneously hybridized on the metaphase plate according to Pauciullo et al. [17], with the exception of the second FISH round in which 3 probes (2p, 2q and 18) were used simultaneously. The labeled probes were mixed and each precipitated in absolute ethanol together with 10 µg salmon sperm DNA and 10 µg calf thymus DNA (both from Sigma). The pellets were vacuum-dried and then resuspended in 15 µl hybridization solution (50% formamide in 2X SSC+10% dextran sulfate) for 1 h at 37°C. The probe solutions were denatured for 10 min at 75°C and pre-hybridized for 60 min at 37°C.

Metaphase preparations were denatured for 3 min in a solution of 70% formamide in 2X SSC (pH 7.0) at 75°C. The slides were hybridized in a moist chamber at 37°C overnight. After hybridization, coverslips were removed by a gentle washing step in 2X SCC. The slides were then washed 3×4 min in washing solution (50% formamide in 2X SSC) at 42°C, followed by 3 additional washing steps for 4 min in 2X SSC at 42°C. Slides were counterstained with DAPI (4,6-diamidino-2-phenylindole) solution (0.24 µg/ml; Sigma) in Antifade (Vector Lab).

The slides were observed at 100x magnification with a Leica DM5500 fluorescence microscope equipped with DAPI, FITC, Spectrum orange specific filters, the FITC/Spectrum orange double filter, and provided with a Cytovision MB 8 image-analysis system (Leica Microsystems, Wetzlar, Germany). Digital images were captured in gray-scale, whereas false colours were created by the image-analyzing system for a reliable evaluation of the painting probes. Approximately 25–30 metaphases were acquired for each slide.

At the end of each round of FISH, the oil for microscope observation was removed from the coverslips and the slides were washed 2×15 min in PBST in a gently shacking, then air dried and immediately reused in the denaturation step for the next round of FISH.

## Results and Discussion

Thirteen chromosome-specific painting probes, generated from river buffalo metaphases via chromosome microdissection and the DOP-PCR procedure were sequentially hybridized on river buffalo, cattle, sheep and goat metaphases in a multi-colour zoo FISH experiment.

**Figure 1:**
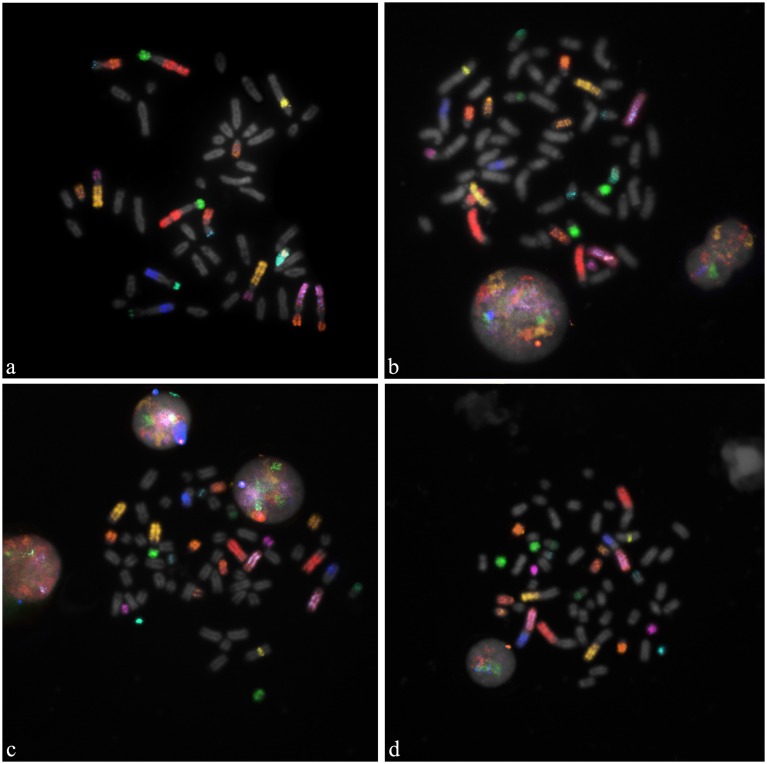
Sequential multicolour hybridization with 13 river buffalo DNA probes on normal males metaphase spreads. Specific signals were clearly detected on: a) river buffalo (*Bubalus bubalis*, 2n = 50) mitosis used as control; b) cattle (*Bos taurus*, 2n = 60); c) goat (*Capra hircus*, 2n = 60) and d) sheep (*Ovis aries*, 2n = 54) mitosis in Zoo-FISH experiments.

**Figure 2:**
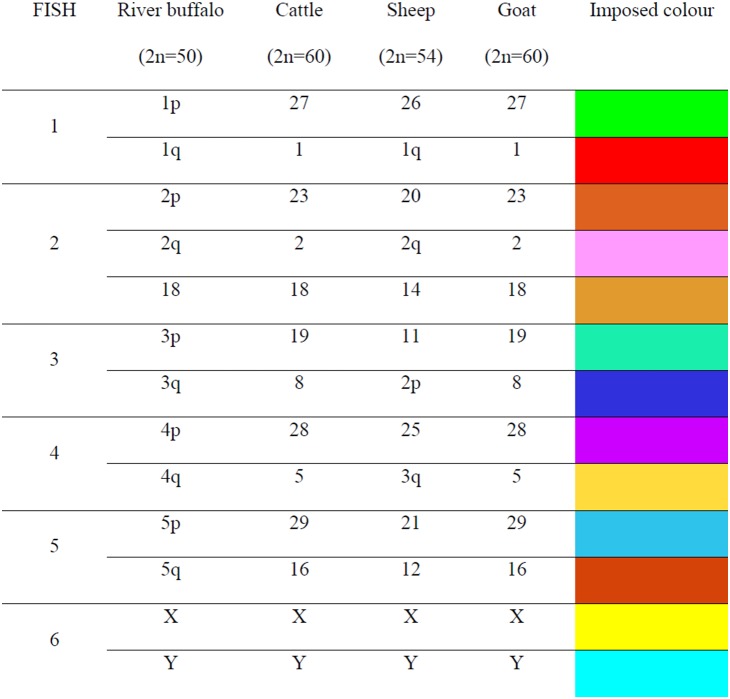
Round of FISH, corresponding homologous chromosomes in river buffalo, cattle, sheep and goat (from ISCNDB, 2000) and super imposed colour for the 13 chromosome/arm specific painting probes.

**Figure 3:**
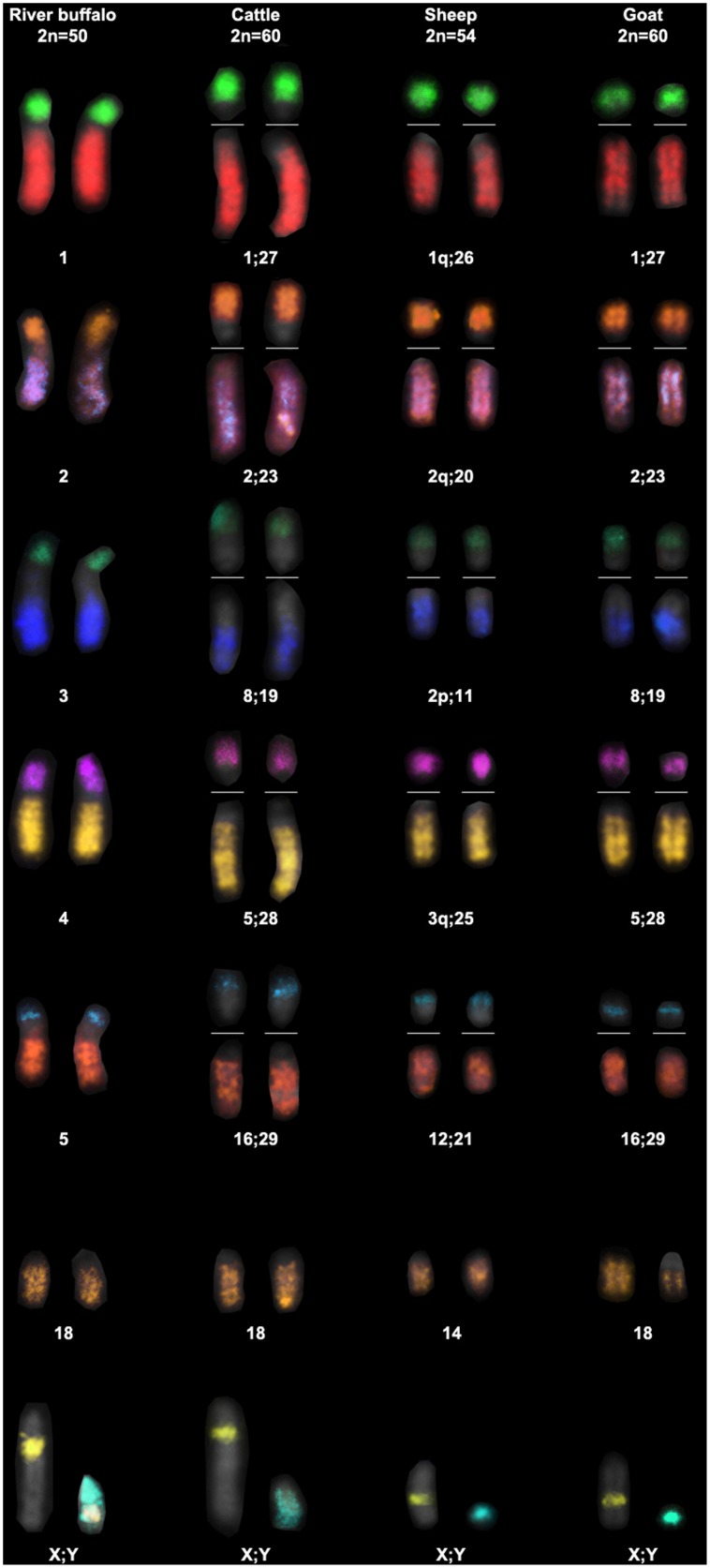
Comparative M-FISH karyotypes generated from the metaphases of the**figure 1**. River buffalo was taken as reference to build the partial karyotype limited to 13 DNA probes. Homologous chromosomes show the same colour among the investigated species. The buffalo Y-chromosome shows a hybrid signal (white) as result of the cross-hybridization of two sex painting probes. This chromosomal band corresponds to the pseudo-autosomal region.

Typically 25–30 metaphase spreads per species were analysed. The DNA probes showed excellent specificity on buffalo mitosis, and the cross hybridization revealed very clearly FISH painting signals in the metaphases and interphase nuclei of all the investigated species (Figure 1). Eight pairs of chromosomes corresponding to the 5 sub-metacentric, two gonosomes and chromosome 18 were simultaneously identified in river buffalo. The same set of probes in the cross-species hybridization experiments covered nearly half of the bovine and goat karyotypes (13 out 30 chromosome pairs), and 40% of the sheep karyotype (11 out of 27 chromosome pairs). These results are summarized in Figure 2. Although this set of probes only partially covers the chromosomal make-up of the investigated species, it allowed the first comparative M-FISH karyotype within the domestic ruminants to be developed (Figure 3).

The chromosomal comparison of different species and the detection of similarities between them is not new. For example, extensive comparative studies have taken place between human and cattle [19], whereas painting probes prepared from flow-sorted chromosomes and made available from the laboratory of Ferguson-Smith have been used in comparative studies in a number of species including human, mouse, pig, cattle, dog, lemurs, Indian and Chinese muntjacs, brown brocket deer, chicken, etc…[13]. Correlation between cytogenetic and gene mapping data is amply shown within the Bovidae (cattle, sheep, goat and buffalo), where similarities in banding patterns are a strong indication of homology at the DNA level [20]. However, sometimes banding patterns can be of little consequence even though the species may belong to the same family (e.g. the species in the family Equidae). The comparative chromosome painting has proved to be an ideal alternative to bypass these problems [13] and several misleading conclusions from earlier Giemsa banding have been refuted by cross-species painting. For example, the conclusion that nucleolus-organizing chromosomes were shared between lesser apes and Old World monkeys was found to be incorrect [21]. The molecular resolution of complex karyotypes by the use of FISH is very helpful within the domestic bovids. This is largely true for the small chromosomes, whose similarity in the banding patterns makes their identification difficult, like those pointed out by the changes in chromosome nomenclature [22], [23], [24] before the approval of the International standards [25].

The use of the FISH with chromosome-specific probes removes any ambiguity in chromosome identification and improves the accuracy of diagnosis for Robertsionan translocations, fusions and more difficult structural rearrangements like reciprocal translocations and inversions. For example, the Robertsonian translocations in the cattle karyotype (2n = 60 with all acrocentric autosomes) might be easily detected by the classical banding methods, in a similar manner to the discovery of the first rob (1; 29) [26]. However, the use of painting probes, BAC probes and molecular markers resolved more complicated cases like the revision of rob (6; 8) and rob (26; 29) [27], or the recent identification of two new rob(14; 17) and rob(21; 23) translocations [28], [29].

The use of classical banding techniques also complicates the identification of chromosomes involved in the fusions (especially in the case of small acrocentric chromosomes), as well as the detection of reciprocal translocations. Bovine whole chromosome paintings were instead successfully used for the identification of a rcp (2; 5) in a mosaic pattern in the Blonde d’Aquitaine breed [30] and recently a rcp (2; 4) (q45; q34) was detected in an Ayrshire bull by Switonski et al. [31]. Inversions are generally complicated to be detected by whole chromosome paintings. Although pericentric inversions could be revealed if appropriate arm specific probes are used, the combination with direct banding would be preferable to maximize the cytogenetic information obtainable [4]. Furthermore, the utility of M-FISH can be also extended to the detection of cryptic aberrations, which is routinely problematic for the animal cytogeneticists, such as telomeric translocations, interstitial deletions, duplications, etc…

The chromosomal identification by classical cytogenetic methods is not suitable also in the analysis of the meiotic metaphases, whose arrangement is usually evaluated in relation of numerical abnormalities. In this regard, the use of whole chromosome paintings in multicolour experiments became extremely useful in the application of preconception genetic diagnosis procedure for the prediction of chromosomal aneuploidies in human secondary oocytes [32].

With the same rationale, the estimation of aneuploidy in the oocytes of the various domestic species and breeds can be considered as an essential step for improving the *in* *vitro* production of embryos destined for the embryo transfer industry, as well as for monitoring future trends of the reproductive health of domestic animals in relation to management errors and/or environmental hazards. In this perspective, the complete set of probes herein produced (with the obvious exception of the Y-probe) was hybridized on 20 bovine secondary oocytes matured *in* *vitro* to test their potential use for aneuploidy detection. The interpretation of the results is based on the consideration that the first polar body (PB I) is the mirror image of the secondary oocyte metaphase (MII), therefore the lack of any chromosome in the MII (nullisomy) has its counterpart in the corresponding PB, which therefore results disomic and viceversa.

**Figure 4:**
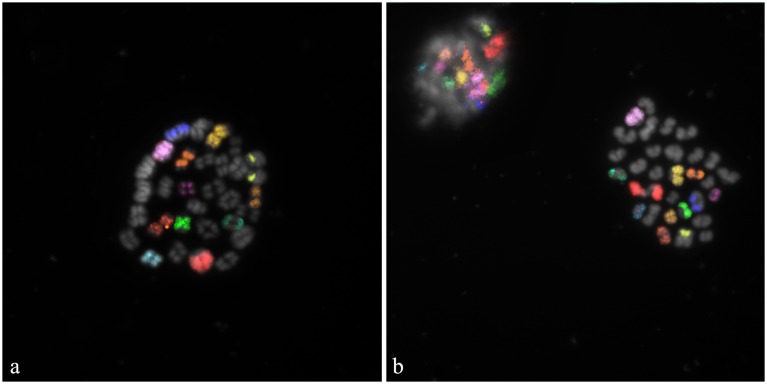
M-FISH carried out on bovine *in* *vitro* maturated secondary oocytes. Specific fluorescent signals were identified on: a) oocyte at the diakinesis/metaphase I stage of the meiosis; b) oocyte at MII and corresponding PB I. Correct chromosomal segregation can be clearly indicated for 11 autosomes and X chromosome.

Specific fluorescent signals were clearly identify for each chromosome in all investigated oocytes. Two out of 20 oocytes showed the presence of bivalents (Figure 4a). Although the pairing of the autosomal bivalents and the sex chromosomes is normal, the occurrence of tetrads and the absence of the corresponding PB I reveals the interruption of the *in* *vitro* maturation at the diakinesis/metaphase I stage of the meiosis (Figure 4a).

The remaining 18 oocytes underwent a normal meiotic division. Specific fluorescent signals were visible on both MII and corresponding PB I (Figure 4b), thus evidencing the correct chromosomal segregation and therefore the lack of abnormalities for the investigated oocytes.

The scarce availability of commercially available chromosome-specific probes -in domestic ruminants- is a limiting factor for the investigation of aneuploidy rates by FISH. This is particularly evident in cattle where only few chromosomes were investigated and few studies are available so far [33], [34]. In addition, the application of other molecular methods like the comparative genomic hybridization (CGH) is not suitable for the analysis of bovine aneuploidies. In fact, differently to what observed in pig [35], the acrocentric nature of cattle autosomes hampers the chromosomal identification after CGH hybridization. As consequence, in this species the identification of specific gains/losses of chromosomal DNA can be detected only by FISH.

Although no abnormalities were detected in the investigated oocytes, these data confirm the potential use of the river buffalo probes for aneuploidy detection in germ cells, thus opening further opportunity of investigation for clinical cytogenetic applications also in the other species with difficult karyotype.

## Conclusions

A DNA collection made of 13 probes generated by chromosome microdissection and DOP-PCR was sequentially hybridised on river buffalo, cattle, sheep and goat metaphase spreads in cross-species hybridization experiments. Nearly half of the bovine and goat karyotypes (13 out 30 chromosome pairs), and 40% of the sheep karyotype (11 out of 27 chromosome pairs) were covered. This allowed the development -for the first time- a comparative M-FISH karyotype for the domestic bovids, which represents a fundamental step for the future achievement of: a) health screening programs of the breeds (highly productive, endangered, indigenous, etc…) related to these species on a molecular cytogenetic basis; b) rapid identification of simple and complex chromosomal rearrangements; c) cross-species hybridization experiments within the family Bovidae and more generally, for comparative evolutionary studies with species of other families; d) resolution of complex karyotypes with particular regard to the detection of hybrid animals; e) evaluation of the aneuploidy level in germ cells as tool for the monitoring the reproductive health of animals in relation to management errors (hormonal imbalances, nutritional and diet mistakes) and/or environmental hazards (mutagens, mitotic poisons) which are known to damage the mitotic/meiotic machinery of the cell.
